# Tirofiban shows better platelet inhibition in diabetic patients during PCI procedures

**Published:** 2010-06

## Introduction

The extent of platelet aggregation and levels of C-reactive protein (CRP) released during actual percutaneous coronary intervention (PCI) procedures have not been well studied.

In this recently published randomised, double-blind study of consecutively eligible patients, rapid-function platelet assays were used to measure platelet aggregation, together with simultaneous measurement of CRP, to compare the efficacy of platelet inhibition of tirofiban and abciximab. Clinical endpoints of the study were death, non-fatal MI, target vessel revascularisation (TVR) with coronary artery bypass grafting or PCI within 30 days of the procedure.^1^

Tirofiban showed greater platelet inhibition in the diabetic patients at the first time point within the PCI procedure. Overall platelet inhibition was similar with the two agents, but there was a trend towards less effective platelet inhibition with abciximab.

Interestingly, measurement of the inflammatory marker, CRP, was similar with both drugs, but these measurements were characterised by a wide variability during the procedure. However, hs-CRP demonstrated an inverse relationship with platelet inhibition over time. The clinical outcomes in the two groups were similar but the small numbers of patients limited the definitive assessment of any difference in the clinical impact of these agents.

The study medications were administered as a bolus plus infusion for two hours, together with heparin, to achieve a target activated clotting time of 200–250 sec/i. Abciximab was dosed as 0.25 μg/kg bolus given immediately before the PCI, followed by 0.125 μg/kg/min (max 10 mg) for 12 hours. A 10-μg/kg bolus of tirofiban was given, followed by a 0.15- μg/kg/min infusion for 12 hours.

This ‘real-world’ situation, although without the recently recommended 25-μg/kg bolus dose of tirofiban, points to the value of achieving steady state before beginning PCI and maintaining this over time. This study was the first to assess CRP levels during a PCI procedure, and an inverse relationship was seen with levels of hs-CRP to platelet inhibition.

## References

[1] Saltzman AJ (2010). The relative effects of abciximab and tirofiban on platelet inhibition and C-reactive protein during coronary intervention.. J Invas Cardiol.

